# Teaching referral skills to medical students

**DOI:** 10.1186/s13104-015-1369-4

**Published:** 2015-08-26

**Authors:** Victoria Bradley, Benjamin C. Whitelaw, Dan Lindfield, Richard J. W. Phillips, Corinne Trim, T. A. Lasoye

**Affiliations:** Department of Post Graduate Medical and Dental Education, King’s College NHS Foundation Trust, London, SE5 9RS UK; King’s College Hospital NHS Foundation Trust, London, SE5 9RS UK; Royal Surrey County Hospital, Egerton Road, Guildford, Surrey, GU2 7XX UK; GKT School of Medical Education, King’s College London, Henriette Raphael Building, London, SE1 1UL UK; Emergency Medicine, King’s College Hospital NHS Foundation Trust, London, SE5 9RS UK

**Keywords:** Referral, Consultation, Medical student, Confidence, Teaching

## Abstract

**Background:**

Referrals are an important and frequent part of a junior doctor’s work. Difficulty with making successful referrals is also very common. Despite this, training in referral skills is not routinely carried out in medical schools.

**Results:**

We designed and delivered a 1-h interactive lecture to final year medical students to teach referral skills. The lecture was delivered on six occasions to up to 70 students at each session. 191 students attended and provided evaluation. 68 % of students had no previous training in referral skills and 99 % felt that referral skills should be included in the undergraduate curriculum. 90 % reported that the lecture had improved their understanding of referral techniques and 83 and 80 % felt that the lecture had improved their ability and confidence, respectively.

**Conclusions:**

Referral skills can be successfully taught in a large group lecture setting. We recommend that the teaching of referral skills is incorporated into all medical schools’ curricula.

## Findings

Referrals are a communication process with the purpose of directing a patient to an appropriate specialist for advice, assessment or admission, and are an essential aspect of management for some patients [1]. This paper concerns verbal referrals only. Emergency departments require their doctors to refer 20–40 % of their patients either for an opinion or for admission [1] and making a referral is recognised to be difficult and stressful [2–4]. Inappropriate, delayed or unsuccessful referrals can lead to adverse patient outcomes and therefore referral is an important aspect of ensuring patient safety [1, 5].

Despite this, referral skills are inconsistently taught to medical students. A recent survey of newly qualified doctors at a UK teaching hospital showed only 43 % (18/42) identified themselves as having received any previous training in how to make a referral [6]. This cohort of doctors had, between them, attended 12 different UK medical schools.

Here we describe and evaluate the effect of an intervention to meet this need by providing teaching for final year medical students on referral skills. We also attempt to quantify the training they have previously received.

Our faculty had previously delivered referral skills training with newly qualified doctors which had been performed in small group tutorials of no more than five participants. That training was based on group discussion and built on candidate experiences. There was not sufficient resource to provide such faculty intensive small group sessions for final year medical students and so the content of the previous work was adapted for use in this larger group in lecture form with the aim of maintaining engagement and discussion.

We designed and delivered a 1-h interactive lecture to final year medical students to teach referral skills. In order to offer teaching to the whole year of 420 students, the lecture was delivered six times to groups of up to 70 students. Maintaining interactivity in such a large group was encouraged by a lecturer who moved off the podium and asked targeted questions of the students.

The content of the lecture included:

1. Introduction to the topic and sharing of previous referral experiences from student candidates.
2. The principles of a good referral which were defined by the faculty by literature review and iterative discussion [PASC]:
  *Preparation* Knowledge of history, examination & key investigations.
  *Agenda recognition* Consider the preferred outcome from the referral, and the other person’s perspective.
  *Structure* To ensure the referral is concise but covers important material.
  ISBAR (Identify, situation, background, assessment & recommendation) is one possible structure. Another structure is a referral proforma script such as the one proposed by Go and colleagues [7].
  *Clarity in communication* State clearly what you want while being polite, clear, helpful, concise and avoid condescension.
  Consider using “magic phrases” [2] such as:
  “I would like you to come and see this patient and give me your opinion”.
  “I believe this patient requires admission under your care”.
  Avoid vague terms such as
  “..can I just run something by you”.
3. Applying this to a scenario. A manufactured scenario was designed based on faculty experience of challenging referrals and detailed to the student audience. Students were asked to assume the role of FY1 who needs to make a referral and shown simulated case notes on the screen. The lecturer facilitated a whole group discussion of the process required to prepare for and the details of the subsequent referral they would make.

Students were then shown a pair of video clips. These were made in advance by the faculty and featured two faculty members in the role of referee and referrer. They show the referral required in the simulated case done well following the above principles, and also done poorly. These videos were then critiqued with the students.

An evaluation form was given to the students to identify their previous experience of training in referral skills, if any. The form asked the students for their self-evaluated level of knowledge and confidence before and after the training. The review of feedback was categorised as service evaluation and thus further ethics approval was not needed.

Evaluation was obtained from 191 students. The data set supporting the results of this article is included within the article. The results demonstrate that 68 % (130/191) had received no previous training in referral skills. 99 % (190/191) felt that teaching on referral skills should be part of their curriculum.

90 % (172/191) felt that the lecture had improved their understanding of referral techniques and 83 % (158/191) felt the lecture would improve their ability to make a referral. 80 % (152/191) felt the lecture had improved their confidence at making referrals.

Referrals are important, stressful and difficult [2–4]. Our data demonstrates almost universal recognition and desire amongst current final year students that referral training should be included in the undergraduate curriculum.

In 1998 a study demonstrated that giving students brief instructions and a one page referral proforma (called a worksheet) led to them giving more comprehensive referrals which were more likely to include both a diagnostic impression and a statement as to the perceived urgency of the referral [7]. The authors concluded that the important skill of referral ought to be taught to medical students and junior doctors. In the intervening 17 years it appears that this recommendation has not been widely adopted for UK undergraduate medical students [6].

Our study demonstrates that a 1-h lecture is a practical method of teaching an introduction to referral skills to final year students. The session included the important and evidence based aspects of making a referral [2, 7, 8]. Self-reported knowledge and confidence is clearly increased by this intervention.

Although we expected the final year medical students would have very limited experience of making referrals at the time point training was offered, this was shown to be untrue. The majority of students had experience of good and bad referrals either vicariously through junior doctors they worked with or personally. Sharing these experiences formed the basis of a discussion about the principles of good and bad referral.

Feedback from the students included some requests to follow each lecture with small group tutorials or practical sessions to enable supervised pair practise. This will be incorporated into future teaching and will become a regular part of the curriculum.

One limitation of this study is that it evaluates only self-reported knowledge, ability and confidence of students. Although this is an important outcome we recognise that a more compelling outcome would be an evaluation of the performance of students doing a real or simulated referral. Given the size of the current study, involving nearly 200 students, this was impractical to evaluate with this group. However, an evaluation of referral performance before and after training with a smaller group is the basis of planned further research.

Referral skills are recognised to be an important competence for doctors but are not currently being taught to the majority of medical students. We have shown that a programme to teach referral skills can be successfully adapted for the large group lecture setting of medical school, with scope for further small group consolidation sessions. We recommend that this topic is incorporated into all medical schools.

## Abbreviations

ED: emergency department
ISBAR: identify, situation, background, assessment, recommendation
